# Challenges in returning results in a genomic medicine implementation study: the Return of Actionable Variants Empirical (RAVE) study

**DOI:** 10.1038/s41525-020-0127-2

**Published:** 2020-05-04

**Authors:** David C. Kochan, Erin Winkler, Noralane Lindor, Gabriel Q. Shaibi, Janet Olson, Pedro J. Caraballo, Robert Freimuth, Joel E. Pacyna, Carmen Radecki Breitkopf, Richard R. Sharp, Iftikhar J. Kullo

**Affiliations:** 1 Department of Cardiovascular Medicine, Mayo Clinic, Rochester, MN USA; 2 Center for Individualized Medicine, Mayo Clinic, Rochester, MN USA; 3 Department of Health Sciences Research, Mayo Clinic, Phoenix, AZ USA; 4 Center for Health Promotion and Disease Prevention, College of Nursing and Health Innovation, Arizona State University, Phoenix, AZ USA; 5 Department of Health Sciences Research, Digital Health Sciences, Mayo Clinic, Rochester, MN USA; 6 Department of Internal Medicine, Mayo Clinic, Rochester, MN USA; 7 Department of Health Sciences Research, Biomedical Ethics Program, Mayo Clinic, Rochester, MN USA

**Keywords:** Genetics research, Genetic testing

## Abstract

To inform the process of returning results in genome sequencing studies, we conducted a quantitative and qualitative assessment of challenges encountered during the Return of Actionable Variants Empiric (RAVE) study conducted at Mayo Clinic. Participants (*n* = 2535, mean age 63 ± 7, 57% female) were sequenced for 68 clinically actionable genes and 14 single nucleotide variants. Of 122 actionable results detected, 118 were returnable; results were returned by a genetic counselor—86 in-person and 12 by phone. Challenges in returning actionable results were encountered in a significant proportion (38%) of the cohort and were related to sequencing and participant contact. Sequencing related challenges (*n* = 14), affecting 13 participants, included reports revised based on clinical presentation (*n* = 3); reports requiring corrections (*n* = 2); mosaicism requiring alternative DNA samples for confirmation (*n* = 3); and variant re-interpretation due to updated informatics pipelines (*n* = 6). Participant contact related challenges (*n* = 44), affecting 38 participants, included nonresponders (*n* = 20), decedents (*n* = 1), and previously known results (*n* = 23). These results should be helpful to investigators preparing for return of results in large-scale genomic sequencing projects.

## Introduction

With increasing interest in genome sequencing to improve health outcomes^1^ there is a need for quantitative and qualitative assessment of challenges related to return of results (RoR) in genomic medicine implementation studies^1^. Although the ethical, legal, and social implications of genomic RoR from research studies have been described^2–4^, little is known about the extent to which challenges may be encountered when conducting return of clinical genomic findings in large research cohorts such as biobanks. To address this gap in knowledge, the eMERGE Network is conducting several genomic medicine implementation studies which involve RoR from a targeted sequencing panel at each of the ten Network sites^5^. These eMERGE Network studies straddle the boundary between research and clinical practice, and as such, may offer valuable insights into the RoR process in population scale genome sequencing efforts.

At the Mayo Clinic, an eMERGE Network site, the Return of Actionable Variants Empiric (RAVE) study^5,6^ aimed to characterize challenges related to genomic RoR and help establish basic guidance for RoR in large-scale genomic sequencing projects. Participants were recruited from Mayo Clinic biobanks in Rochester, Minnesota and Phoenix, Arizona on the basis of hypercholesterolemia and/or colon polyps to enrich for familial hypercholesterolemia (FH) and hereditary colorectal cancer—two conditions labeled by the Office of Public Health Genomics, Centers for Disease Control and Prevention^7^ as Tier 1 genomic applications due to the potential for positive impact on public health based on available evidence-based guidelines and recommendations^8^. Pathogenic (P) and likely pathogenic (LP) variants from sequencing of 68 disease related genes and 14 actionable single nucleotide variants (SNVs) were returned to study participants (Supplementary Tables 1, 2). Variants of uncertain significance (VUS), likely benign, and benign variants were considered to be “neutral” findings. In this report, we describe challenges encountered during RoR in the RAVE Rochester, Minnesota cohort. Challenges in returning results in the RAVE Phoenix, Arizona cohort including 500 Latino participants who received care at a Federally Qualified Health Center^9^ are reported separately due to methodological differences in the RoR process at each site.

## Results

**Fig. 1:**
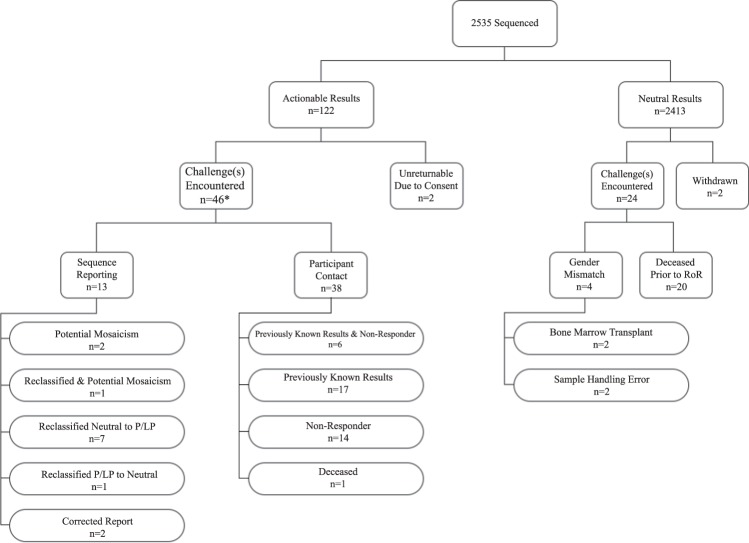
An overview of the challenges encountered during return of results in the RAVE study. Previously known: participants in whom a P/LP sequencing result had already been documented in the EHR subsequent to clinical testing. Non-responder: participants who did not respond to four contact attempts to return a P/LP finding. *5 participants encountered challenges in both sequence reporting and participant contact.

**Fig. 2:**
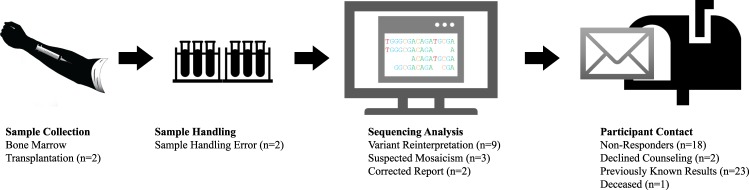
A temporal representation of the challenges encountered during return of results in the RAVE study. Challenges in returning results included those related to sample collection and handling, variant interpretation, and contacting participants.

Participant characteristics are summarized in Table 1. The mean age of the cohort was 63.9 ± 7.7 years and 57% were female. Nearly half (47%) of participants had college or greater education, 64% reported employee provided health insurance and a majority (89%) had adequate health literacy. Of 2535 RAVE participants, 122 (4.8%) had actionable results and 2413 had neutral results. Challenges encountered during the RAVE study are summarized in Figs 1 and 2 and described in detail below.

**Table 1:** RAVE participant characteristics.

	(*n* = 2535)
Age, years	63.9 ± 7.7
Sex, female	1454 (57%)
Race, white	2468 (97%)
Education	
College or greater	1196 (47%)
High School or Some College	1153 (45%)
Health Literacy	
Adequate	2253 (89%)
Inadequate	151 (6%)
Missing	131 (5%)
Insurance	
Employer	1627 (64%)
Government	776 (31%)
Private	242 (10%)
No Insurance	17 (<1%)
Missing	127 (5%)

### Return of neutral results

The vast majority of neutral results (*n* = 2291) were returned to participants by mail; 103 participants had neutral results returned by a genetic counselor (GC) as part of an ancillary study^10^. Challenges in returning neutral results arose in 24 participants including gender mismatches detected by the sequencing laboratory (*n* = 4, 0.17%) and deceased participants (*n* = 20, 0.8%).

Gender mismatches were observed in four of the 2535 samples sequenced. Two discrepancies were attributed to the participant having received a bone marrow transplant from a sibling of the opposite sex prior to having blood samples drawn for the study. These participants were withdrawn from the study and healthcare providers were notified that the sequencing results were not valid. The other two discrepancies appeared to have stemmed from errors in sample handling, and new DNA samples from these latter two participants were sent for sequencing.

Twenty participants with neutral results died prior to RoR. Sequencing results were not returned to families or to primary care providers (PCPs) of these participants; however, results were placed in the electronic health record (EHR).

### Return of actionable results

Of 122 actionable results, two were not returnable due to participant consent choice, one result was not returnable due to participant death and one actionable result was not returned given phenotype–genotype mismatch and subsequent reclassification of the variant from P/LP to a VUS. Of the 118 returnable actionable findings, 86 were returned in-person by a GC and additional 12 results were returned by a GC over the phone due to the participants’ geographic location or adverse weather. Actionable genetic variants related to the CDC Tier 1 conditions were present in 59 participants (2.3% of the overall cohort). These included FH (*n* = 26, 1:98), hereditary breast and ovarian cancer (HBOC) syndrome (*n* = 18, 1:141), and Lynch syndrome (*n* = 15, 1:169). Notably, HBOC variants were detected in this cohort at a greater than expected frequency despite no intentional enrichment of the cohort for the condition.

Broadly, challenges in actionable RoR, encountered in 38% of participants, were related to sequencing reports (phenotype discrepant findings, reports requiring correction, mosaicism, and reclassification) and participant contact (nonresponders or declined genetic counseling, deceased, and previously known results).

Challenges related to sequencing reports included nine results which were reclassified by the sequencing center during the RAVE study. Three results were reclassified after Mayo investigators requested additional review of variants based on clinical features of the participants and six were reclassified by the sequencing center following analyses for structural variants or changes to the analytical pipeline. In addition, two reports required corrections due to erroneous reporting of the variant-disease association.

The study team identified six participants with clinical features of FH^11^ in whom no P/LP variants in FH genes were reported by the sequencing laboratory, one having a known LP variant in *LDLR* documented in the EHR. Findings from literature, sequencing data, and detailed EHR review of these participants were discussed with the sequencing laboratory and two variants were reclassified from VUS to LP with the remaining four designated as VUS ‘leaning pathogenic’. Thereafter the RAVE study team opted to return these results to study participants. In a 70-year-old participant, a duplication of exons 14–17 in *NF2* was labeled P/LP by the sequencing laboratory, indicating Neurofibromatosis Type 2. EHR review revealed no clinical features of neurofibromatosis, literature review revealed no reports of this variant being considered pathogenic and an external laboratory with expertise in neurofibromatosis testing did not consider the variant to be pathogenic. Upon further discussion the sequencing laboratory reclassified the variant from P/LP to a VUS and a neutral report was issued.

Sequencing report errors included a pathogenic variant in *CACNA1S* (c.1583G>A) associated with hypokalemic periodic paralysis^12^, which was detected in a participant sample; however, the sequencing report incorrectly associated this variant with malignant hyperthermia. The participant indeed did have a clinical history of hypokalemic periodic paralysis. In another report, an *SCN5A* variant (c.3956G>T) which has been associated with Brugada syndrome^13^ was described as being associated with Long-QT syndrome. These discrepancies were communicated with the sequencing center and corrected reports were issued prior to RoR.

Mosaicism was suspected in three participants. One male participant with a *TP53* variant (c.578A>T) had a history of chronic lymphocytic leukemia. During RoR the GC recommended germline confirmation of the variant via a tissue sample. A female participant had a truncating *APC* variant (c.1262G>A, p.Trp421*) deemed pathogenic for familial adenomatous polyposis. This variant was identified with a low allele fraction and required co-amplification at lower denaturation temperature-polymerase chain reaction for enrichment. The participant had a normal colonoscopy at age 57 and no family history of colorectal cancer or polyposis. As such this variant was returned to the participant by GC with the caveat that it likely represented somatic or germline mosaicism. Testing of offspring for this genetic variant was recommended. A third report indicated a total deletion of exons 1–27 of *BRCA2* and exons 1–27 of *RB1* indicating increased risk of breast cancer and retinoblastoma, respectively. The sequencing report suggested that these deletions may have been observed due to a larger deletion on chromosome 13q and that the sequencing analysis and multiplex ligation-dependent probe amplification suggested a somatic deletion. The participant was a 66 year old male with no phenotypic manifestation related to either finding.

Six sequencing reports were reissued by the sequencing laboratory after updated variant analysis pipelines; including five resulting from the discovery of structural variants. All were reclassified from neutral to P/LP (Table 2), and of these, four reports had to be reissued to participants who had previously received a neutral report. Two reports were issued for participants who already had clinically documented *MSH2* copy number variations associated with Lynch syndrome^14–16^. Both participants were nonresponders. Similarly, a report was issued for a female participant with a known BRCA1 copy number variant associated with HBOC and the participant completed counseling. Three reclassified reports were issued including a deletion of exons 1–15 of *CHEK2* in a male participant diagnosed with prostate cancer at age 46 with a strong family history of prostate cancer^17^, a male participant with a deletion of both *BRCA2* and *RB1*, and a male participant discovered to be homozygous for Factor V Leiden. These three participants were re-contacted to disclose the updated result and each completed counseling.

**Table 2:** A list of variants that were reclassified to actionable.

Gene	Variant	Neutral result returned to participant?	Time between neutral report and reclassification (days)
*MSH2*^a^	Deletion exons 4–6	Yes	529
*MSH2*^a^	Deletion exons 1–3	Yes	603
*BRCA1*^a^	Deletion exons 13–15	No	38
*CHEK2*	Deletion exons 1–15	Yes	603
*BRCA2*^b^	Deletion exons 1–27	Yes	605
*RB1*^b^	Deletion exons 1–27		
*F5*	Homozygous c.1601G>A	Yes	321

^a^Participant had result documented in the EHR before study related return of results.

^b^Two reclassified variants in a single participant.

Of 118 participants with returnable actionable results, 98 completed genetic counseling; 58 participants responded after one letter, 18 responded after two letters, 17 responded after three letters, and five responded after receiving a fourth letter. Eighteen participants (14%) were nonresponders (i.e., did not respond for four mailed letters) and additional two participants declined to meet with a GC (2%). Results for these 20 participants were placed in the EHR with PCP notification of the result. Nonresponders tended to be younger than responders (*P* = 0.007). Participant characteristics such as gender, insurance status, education, health literacy, and previously known results were not significantly (*P* > 0.05) associated with nonresponse.

Two participants with actionable results died before the study team received their sequencing results. These deceased participants had actionable findings in *PALB2* and *TSC2* indicating breast and pancreatic cancer risk and tuberous sclerosis type II, respectively. The study team pursued return of the *PALB2* variant to a legal representative of the participant. The *TSC2* variant was not returnable as the participant had elected to receive results related only to hypercholesterolemia or colon polyps; however the participant had been diagnosed with tuberous sclerosis while living.

In 23 (19%) RAVE participants with actionable results, sequencing results had already been documented in the EHR with the majority (*n* = 15) being related to the CDC Tier 1 disorders including nine results in *BRCA1* and *BRCA2*, three in *APOB* and *LDLR*, and three in *MSH2* and *MSH6* (Table 3). Results previously documented in the EHR for non-Tier 1 disorders included hereditary hemochromatosis (*n* = 5), long-QT syndrome (*n* = 2), and familial adenomatous polyposis (*n* = 1).

**Table 3:** Participants with actionable variants in Tier 1 genes and the number who had been previously documented to have the variants.

Gene	Participants (*n*)	Result documented in EHR (*n*)
FH genes	26	3
* APOB*	6	1
* LDLR*	19	2
* PCSK9*	1	0
HBOC genes	18	9
* BRCA1*	7	6
* BRCA2*	11	3
CRC genes	15	3
* MLH1*	1	0
* MSH2*	2	2
* MSH6*	3	1
* PMS2*	9	0
Total	59	15

*FH* familial hypercholesterolemia, *HBOC* hereditary breast and ovarian cancer syndrome, *CRC* colorectal cancer.

## Discussion

Several large population scale sequencing projects are underway^1,18–23^ and it is expected that 2–5% of participants in such cohorts will have medically actionable findings in the ACMG 56™ or ACMG 59™ genes^24^. Investigators leading such projects should be aware of the challenges that may arise when returning medically actionable genomic sequencing results. In the present study we report for the first time the burden of such challenges in a large sequencing study. Among participants with neutral results (*n* = 2413) gender mismatch (*n* = 4, 0.17%) and decedents (*n* = 20, 0.8%) were the primary issues encountered. Among participants with actionable results (*n* = 122), challenges were encountered during RoR in a substantial proportion (38 %) of the RAVE cohort occurring primarily in two categories: sequencing reports and interpretation (11%), and participant contact (31%) (Table 4). It is expected that the frequency and occurrence of the challenges encountered in similar studies will vary from those reported here. Factors such as the time elapsed between consent and reporting of sequencing findings, cohort age, demographics, and the health characteristics of the studied population may affect the degree to which the challenges described are encountered and may introduce additional challenges not characterized in this manuscript.

**Table 4:** A summary of challenges encountered in the RAVE study and recommendations.

Challenges	Recommendation
Sequencing reports	
Gender Mismatch	Gender mismatch may be detected, especially in large cohorts. Gender mismatch may occur due to clerical or lab errors, bone marrow transplantation from the opposite sex, or transgenderism. The DNA source used for sequencing (i.e., blood, tissue, etc.) is important. Recent blood transfusion or bone marrow transplantation may cause unintended sample mismatches and should be asked about at the time of enrollment.
Mosaicism	Mosaicism is possible when sequencing DNA from blood cells or saliva and should be considered when there is phenotype-genotype discrepancy. A hematologic malignancy or bone marrow transplantation prior to DNA sampling can contribute to mosaicism. Sample types used for sequencing should be considered accordingly.
Sequencing Discrepancies/Variant Classification	The EHR can provide context and detailed phenotype information for variant interpretation. A patient’s medical and family history can provide meaningful context to the variant interpretation process. Discrepancies should be conveyed to the sequencing site.
Reclassification of Variants	Anticipate the possibility of reclassification of variants. Genetic variants may be reinterpreted as analytical tools evolve and additional knowledge emerges. Participants should be made aware of this possibility when consenting to genomic sequencing studies. Stakeholders should plan to support re-disclosure of sequencing results in the event of variant reclassification and to document amended sequencing findings in the EHR.
Participant contact	
Maintaining Patient/Participant Status	Maintaining up-to-date contact information and vital status on participants is important. Information related to primary care providers, mailing addresses or contact information and vital status should be updated prior to contact attempts.
Non-responders/Refusal to receive counseling	Some patients may not respond to attempts to disclose actionable results or may refuse genetic counseling. Contact materials for participants should be carefully worded, and should provide adequate motivation for follow-up. Consider the use of certified mail for letter tracking and verification of receipt. Consider placing results in the EHR after a certain period of non-response. In RAVE, participant results were placed in the EHR after 6 months of non-response and relevant providers (usually the PCP) were notified electronically by the study genetic counselor or the study investigator.
Deceased participants	Participants may die before results can be returned to them. Actionable genetic findings often carry implications for first degree relatives of deceased participants. It may be helpful to obtain consent to contact a family member or representative with results in the event that a participant dies before receiving their result.
Results already in the EHR	Some participants in large cohorts may have already received genetic testing results. As genetic testing becomes more widely used clinically for diagnosis, some genetic findings may already be clinically known. Expect this possibility and design study materials accordingly.

Delivery of accurate and clinically valid sequencing results is paramount to the practice of clinical genomics. In large scale sequencing projects, laboratories may identify gender mismatches^25^, which may result from transgenderism, allogenic stem cell transplantation, or sample handling/data entry errors. Events such as allogenic bone marrow transplantation, organ transplantation, blood transfusion in proximity to sample collection, or certain malignancies prior to the collection of blood or saliva samples for sequencing can alter or invalidate sequencing results, lead to mosaicism, or may lead to unintentional sequencing of a donor’s germline rather than the intended participant^26,27^. Stakeholders should be sensitive of these possibilities and plan accordingly.

Variant classification and pathogenicity assignment are labor intensive processes and despite established guidelines sequence interpretations may vary^28–31^. Considering clinical context may aid variant classification; however, sequencing laboratories often do not have access or the bandwidth to perform detailed phenotype review in large-scale contexts^32^. Detailed EHR review of participants with actionable results in the present study resulted in reclassification of three variants; two of which were upgraded from VUS to LP, and one which was downgraded from P to VUS. Investigators should consider leveraging detailed phenotype information in the EHR to inform variant interpretation and should be prepared to discuss any discrepancies between observed genotype and phenotype.

Analytical pipelines for assessing sequencing data are constantly evolving, as is the understanding of the functional consequences of variants. With emergence of new knowledge, variants may be reclassified^33,34^. As such, project leaders should plan to support reclassification activities potentially well beyond the initial RoR process. Sequencing reports in the EHR may also need to be amended as variants are reclassified, with updates issued to participants and care providers to enable appropriate follow-up^35^.

Information related to mailing addresses or contact information and vital status should be updated prior to contact attempts. Despite best efforts to return results, some participants may not respond to contact attempts and results may have to be placed in the EHR. Participants who consent to receive genetic test results should be informed that results will be placed in the EHR in case of nonresponse. In the RAVE study, results were placed in the EHR with PCP notification after 6 months of nonresponse to contact attempts so appropriate clinical intervention could be pursued at the discretion of the provider. The ability to predict nonresponse in large sequencing cohorts could be helpful when preparing for RoR. In the RAVE cohort, younger age but not insurance status, health literacy, or education level were associated with nonresponse. Studies of larger cohorts, however, are warranted as our study sample may not be powered to detect such associations.

Genomic RoR studies should consider the possibility of participant death and have mechanisms in place to facilitate RoR to at-risk relatives in such situations^36–38^. Biological relatives share risk of carrying the same pathogenic variant as the study participant. Informed consent should allow disclosure of sequencing results to a legal representative or biological relatives in the event that a participant dies. Studies exploring genomic RoR in the context of participant loss of capacity to receive results or death^36^ support consent materials which provide a clear path for investigators to respect the decisions of deceased participants^37^. A toolkit has been developed to promote sharing of genomic research results with relatives of living or deceased research participants^38^. In the present study we did not inform family members or a legal representative of deceased participants with neutral results, but results were documented in the EHR. The RAVE study team did pursue the disclosure of an actionable finding to a legal representative of a deceased participant.

As genetic testing becomes more widely used to assess risk of disease, some genetic findings discovered in research studies may already be clinically known. Investigators should be aware of this possibility and design protocols and materials accordingly to potentially avoid redundant genetic testing and counseling. In the RAVE study nearly a fifth of participants with actionable results (19%) already had the actionable result documented in the EHR. Of the 58 Tier 1 actionable results identified in RAVE 15 were previously documented in the EHR—nine HBOC related results, three FH results, and three hereditary colorectal cancer results. Conversely, knowing a particular genetic test results for a condition does not preclude an individual from having additional actionable results; three RAVE participants had multiple actionable results detected by sequencing. Alerting providers about the availability of neutral results from a genomic medicine study may prevent duplicative genetic testing in their patients.

The EHR is an important resource for implementing genomic medicine and the RAVE study was intentionally EHR-based^35^. One goal of RAVE was to integrate genomic results in the EHR beyond simply placing a PDF of the report in the EHR. XML files were used to generate alerts for PCPs; at the beginning of RoR an XML parser inadvertently sent several alerts to PCPs prior to the participant-GC encounter. Thus EHR-based genomic implementation studies will also need to consider the challenge of integrating results in the EHR with linkage to clinical decision support to guide PCPs at the point of care.

A strength of this study is the quantitative and qualitative evaluation of challenges in returning results in a genomic medicine implementation study conducted in a single academic medical center. Several eMERGE network projects are ongoing to examine challenges across a variety of study settings and participant populations, providing diverse perspectives regarding RoR from sequencing studies. Challenges related to genomic RoR to an underserved minority community at a federally qualified health center, psychosocial response to RoR and outcomes consequent to RoR are described in separate reports. This study did not address additional categories of results obtainable from genome sequencing such as polygenic risk scores, pharmacogenomic results, carrier status, and ancestry, which may present additional challenges from ethical, legal, and practical standpoints. Another limitation is that the methods we used to quantify barriers related to genomic medicine implementation may not have identified all potential challenges that could be encountered and certain challenges may be overrepresented in this study. As such, the observations from this study warrant replication in other large-scale efforts.

In a genomic medicine implementation study, challenges in returning neutral results were encountered in ~1% of participants and were related to gender mismatches and deceased participants. In contrast, challenges were encountered in 38% of participants with actionable results and were primarily related to sequencing reports and participant contact. Our findings should be helpful for researchers and health systems overseeing large scale genomic sequencing projects^39^, enabling them to be better prepared for returning results by anticipating and perhaps even pre-empting some of the challenges described herein.

## Methods

The design and initial results of the RAVE study have been previously described^6^. In brief, 2535 participants from Mayo Clinic biobanks in Rochester, Minnesota who had hypercholesterolemia, colon polyps, or both, underwent targeted diagnostic sequencing of 68 genes and 14 SNVs using the eMERGEseq panel at Baylor College of Medicine Human Genome Sequencing Center, a Central Laboratory Improvement Amendment certified facility. Sequencing results were validated by Sanger sequencing, and quality control was performed using Fluidigm SNPTrace array and Illumina Human Core exome arrays to assess whether gender-allocation mismatches or procedural errors occurred during preparation, transfer, or sequencing of participant samples.

### Enrollment

Participants were recruited by mail from two Mayo Clinic Rochester biobanks. Participants were as follows: (1) residents of Southeast Minnesota who were alive and aged 18–70 years; (2) had low-density cholesterol (LDL-C) ≥155 or ≥116 mg/dL on lipid-lowering therapy with no known cause of secondary hyperlipidemia, or a personal history of colon polyps documented in the EHR; and (3) had no cognitive impairment or dementia that would compromise the candidate’s ability to provide written informed consent. Genetic counseling and telephone support was available to participants who had questions about the RAVE study^40^. Those who elected to participate in RAVE returned the informed consent document with their signature (*n* = 2535) and an accompanying questionnaire (*n* = 2442).

### Informed consent

This study was approved by the Mayo Clinic institutional review board (IRB). To participate, individuals consented to receive primary findings (i.e., those related to hypercholesterolemia or colon cancer risk) but could opt out of receiving secondary findings (i.e., related to other medical indications). Participants could withdraw from the study at any time prior to the disclosure of results. The 68 disease related genes on the eMERGEseq panel included the ACMG 56™ genes^41^, an additional 12 genes, and 14 SNVs deemed medically actionable by eMERGE investigators. Additional details regarding the informed consent process for the study have been previously described^5^.

### Demographics

Demographic information including age, sex, ethnicity, and race were extracted from the EHR. Education level, health literacy^42^, and insurance status were obtained from study questionnaires.

### Return of results

RoR began ~2 years after participant consent. Participants with neutral results (i.e., no actionable variants identified) were notified of their results by postal mail (except for 103 participants who received results from a study GC as part of an ancillary study^10^) but had the option of speaking with the study team or a GC by telephone to discuss their results. Sequencing reports were scanned into the EHR and an Epic® in-basket message summarizing the results was sent to PCPs.

Participants with actionable results were mailed a letter stating that RAVE-sequencing results were available and that the participant should contact the study team to schedule an appointment with a GC. The original protocol allowed up to three mailed letters to schedule appointments; however, the study team was granted IRB approval for a fourth, more strongly worded contact letter to motivate participants who had not responded to the initial three letters to contact the study team. In addition, the protocol for mailed contact was modified during the study to enable the use of certified mail to track the receipt of study contact materials. Participants with actionable results who did not respond to four contact attempts were considered to be non-responders. Results for nonresponders were placed in the EHR and the participant’s PCP or a medical provider determined to be the most contextually appropriate was notified of the result via an Epic® in-basket message. One of the three study GCs disclosed results to each participant who responded. Genetic counseling included a brief summary of the study, education about sequencing results, potential implications for medical management, risks to family members, and referral to an appropriate specialist or department for further clinical management. Participants who resided in a state where study GCs had licensure were eligible to receive results from the GC over the telephone.

### Placement of genomic results in the EHR

Sequencing results were reported to the study team in multiple formats including PDF, HTML, JSON, and XML files templated on a schema developed by the eMERGE network based on Health Level 7 version 2 Genetic Test Results standards^43^. XML files were used to generate messages for PCPs based on sequencing results. Messages were reviewed and personalized by study GCs before transmittal to the PCP.

### Documentation of challenges

Documentation of study related challenges included evaluation of correspondence from three study GCs, correspondence with the sequencing site, meeting minutes and notes from bimonthly investigator meetings, communication with the IRB, correspondence from nonstudy clinicians, and review of study tracking documentation. Study coordinators maintained a study log which documented contact attempts, genomic-sequencing results, study team correspondence, participant demographics, participant vital status, enrollment status, and EHR information relevant to sequencing results. Study documentation was analyzed to generate a summary of the challenges that were encountered during RoR. Challenges in returning actionable results were grouped into two categories: those related to sequence reporting, and those related to participant contact.

### Data management and statistical methods

Study data were maintained on a secure, password protected, and institutionally hosted server available only to study personnel. Evaluations of the data included general inspection of the raw data, plots of summary scores, examination of outliers and group distributions, and evaluation of missing data. Differences in participant characteristics between responders and nonresponders were evaluated by *t*-test for continuous traits and by chi-square test for dichotomous traits, using the statistical software JMP®, Version 14 (SAS Institute Inc., Cary, NC, 1989–2019).

### Reporting summary

Further information on experimental design is available in the Nature Research Reporting Summary linked to this article.

## Supplementary information

Reporting Summary

Supplementary Information

## Data Availability

The datasets generated during and/or analyzed during the current study are available from the corresponding author on reasonable request. Sequencing data generated during eMERGE Network Phase III, including the RAVE study, is available is available on dbGaP (Accession: phs001616.v1.p1).
