# Supplementary material for: Challenges in returning results in a genomic medicine implementation study: the Return of Actionable Variants Empirical (RAVE) study
Source: NPJ Genom Med. 2020 May 4;5:19. doi: 10.1038/s41525-020-0127-2 (PMC7198538; doi:10.1038/s41525-020-0127-2)
Supplement: Supplementary file 2 — Supplementary Information [file 41525_2020_127_MOESM2_ESM.pdf]

Supplementary Material

Supplementary Table 1. eMERGEseq consensus gene list		
**Category**	**Phenotype**	**Gene/genes**
**Cardiovascular**	Ehlers-Danlos syndrome, vascular type	*COL3A1, COL5A1*
	Marfan syndrome, Loeys-Dietz syndromes, and familial thoracic aortic aneurysms and dissections	*FBN1, TGFBR1, TGFBR2, SMAD3, ACTA2, MYH1, MYLK*
	Hypertrophic cardiomyopathy	*MYBPC3, MYH7, TNNT2, TNNI3, TPM, MYL3, ACTC1, PRKAG2, GLA*
	Dilated cardiomyopathy	*MYL2, LMNA*
	Catecholaminergic polymorphic ventricular tachycardia	*RYR2*
	Arrhythmogenic right ventricular cardiomyopathy	*PKP2, DSP ,DSC2, TMEM43, DSG2*
	Romano-Ward long-QT syndrome types 1, 2, and3, Brugada syndrome	*KCNQ1, KCNH2, SCN5A, KCNJ2, KCNE1*
	Familial hypercholesterolemia	*LDLR, APOB, PCSK9*
	Diabetes	*HNF1A, HNF1B*
**Cancer**	Hereditary breast and ovarian cancer	*BRCA1, BRCA2,*
	Li-Fraumeni syndrome	*TP53*
	Peutz-Jeghers syndrome	*STK11*
	Lynch syndrome	*MLH1, MSH2, MSH6, PMS2, POLD1, POLE*
	Familial adenomatous polyposis	*APC*
	MYH-associated polyposis; adenomas, multiple colorectal, *FAP* type 2; colorectal adenomatous polyposis, autosomal recessive, with pilomatricomas	*MUTYH*
	Juvenile polyposis	*BMPR1A, SMAD4*
	Von Hippel–Lindau syndrome	*VHL*
	Multiple endocrine neoplasia type 1	*MEN1*
	Multiple endocrine neoplasia type 2	*RET*
	Familial medullary thyroid cancer[d]	*RET*
	PTEN hamartoma tumor syndrome	*PTEN*
	Retinoblastoma	*RB1*
	Hereditary paraganglioma-pheochromocytoma syndrome	*SDHD, SDHAF2, SDHC, SDHB*
**Other**	Tuberous sclerosis complex	*TSC1, TSC2*

	WT1-related Wilms tumor	*WT1*
	Neurofibromatosis type 2	*NF2*
	Wilson disease	*ATP7B*
	Ornithine transcarbamylase deficiency	*OTC*
	Neuromuscular disorders	*RYR1, CACNA1A, CACNA1S*

Supplementary Table 2. Consensus list of actionable single nucleotide variants to be returned.

rs#	Gene	Associated Disease	Disease Category
rs77931234	*ACADM*	Medium-chain acyl-CoA dehydrogenase (MCAD) deficiency	Inborn error of metabolism
rs387906225	*ALDOB*	Hereditary fructose intolerance	Inborn error of metabolism
rs386834233	*BCKDHB*	Maple syrup urine disease	Inborn error of metabolism
rs79761867	*BCKDHB*	Maple syrup urine disease	Inborn error of metabolism
rs80338898	*FAH*	Tyrosinemia type I	Inborn error of metabolism
rs1801175	*G6PC*	Glycogen storage disease type I	Inborn error of metabolism
rs397509431	*CPT2*	Carnitine palmitoyltransferase II (CPT II) deficiency	Inborn error of metabolism
rs113993962	*BLM*	Bloom Syndrome	Cancer susceptibility
rs193922376	*MSH2*	Lynch syndrome#	Cancer susceptibility
rs6467	*CYP21A2*	21-hydroxylase deficiency	Endocrinology
rs6025	*F5*	factor V Leiden thrombophilia*	Thrombophilia
rs1800562	*HFE*	Hereditary hemochromatosis	Iron storage
rs28940579	*MEFV*	Familial Mediterranean fever	Inflammatory
rs61752717	*MEFV*	Familial Mediterranean fever	Inflammatory

Only bi-allelic (homozygous, or if applicable compound heterozygous) variants were returned. Mode of inheritance is autosomal recessive except #=autosomal dominant and *=risk increased.
